# Emergency Department Admissions Among Older Adults Living Alone With Multimorbidity

**DOI:** 10.1093/geroni/igab046.2226

**Published:** 2021-12-17

**Authors:** Jon Barrenetxea, Cynthia Chen, Woon-Puay Koh, Feng Qiushi, Kelvin Bryan Tan, Kevin Chua, Rachel Tong

**Affiliations:** 1 Duke-NUS Medical School, Singapore, Not Applicable, Singapore; 2 National University of Singapore, National University of Singapore, Not Applicable, Singapore; 3 Ministry of Health, Singapore, Singapore, Not Applicable, Singapore

## Abstract

Older adults living alone are at higher risk of mortality, morbidity and healthcare utilization. As more older adults live alone, Emergency Department (ED) admissions could rapidly increase, particularly among those with multimorbidity. We studied the association of living alone on ED admissions among older adults with multimorbidity. We used data from 16,785 older adults of the population-based Singapore Chinese Health Study (mean age: 73 years, range: 61-96 years) who were interviewed in 2014-2016 for living arrangements and medical history. Participants were followed-up for one year on ED admission outcomes (number of admissions, inpatient days and hospitalization costs). We used multivariable logistic regression to study the association between living alone and ED admission, and ran two-part models (probit & generalised linear model) to estimate the association of living alone on inpatient days and hospitalization cost. We found that compared to living with others, living alone was associated with a higher odds of ED admissions [Odds Ratio (OR) 1.28, 95% Confidence Interval (CI) 1.08-1.51)], longer inpatient days (+0.61, 95% CI 0.25-0.97) and higher hospitalization costs (+322 USD, 95% CI 54-591). Compared to those living with others without multimorbidity, living alone with multimorbidity was associated with higher odds of ED admission (OR 1.64 95% CI 1.33-2.03), longer inpatient days (+0.73, 95% CI 0.29-1.17) and higher hospitalization costs (+567 USD, 95% CI 230-906). In conclusion, living alone is associated with higher odds of ED admission, longer inpatient days and higher hospitalization costs among older adults, particularly among those with multimorbidity.

